# Effect of drug-eluting stents on 1-year risk of new-onset atrial fibrillation in patients with acute myocardial infarction treated with percutaneous coronary intervention

**DOI:** 10.1097/MD.0000000000021885

**Published:** 2020-08-21

**Authors:** Fa-Chang Yu, Ya-Hui Chang, I-Ming Chen, Hung-Yi Liu, Chao-Feng Lin, Li-Nien Chien

**Affiliations:** a Division of Cardiology, Department of Internal Medicine; b Department of Pharmacy, MacKay Memorial Hospital; c Department of Medicine, School of Medicine, National Yang Ming University; d School of Public Health, College of Public Health and Nutrition, Taipei Medical University, Taipei; e Department of Medicine, MacKay Medical College, New Taipei City; f Ph.D. Program for Cancer Molecular Biology and Drug Discovery, College of Medical Science and Technology, Taipei Medical University and Academia Sinica; g School of Health Care Administration, College of Management, Taipei Medical University, Taipei, Taiwan.

**Keywords:** acute myocardial infarction, atrial fibrillation, drug-eluting stent

## Abstract

The use of a drug-eluting stent (DES) in patients with acute myocardial infarction (AMI) treated with percutaneous coronary intervention is conventional. However, the effect of DES on new-onset atrial fibrillation (AF) after AMI still remains unclear.

By using data from Taiwan's National Health Insurance Research Database, a total of 17,741 patients with ST-elevation myocardial infarction (STEMI) and 17,631 patients with non-ST-elevation myocardial infarction (NSTEMI) treated with percutaneous coronary intervention were analyzed to investigate the risk of new-onset AF after index admission of AMI.

There were 26.5% (N = 4696) of patients with STEMI and 39.5% (N = 6967) of patients with NSTEMI received DES implantation. Upon 1-year follow-up, we observed that DES placement was associated with a reduced 1-year risk of new-onset AF in the patients with NSTEMI (adjusted hazard ratio [aHR] = 0.74, 95% confidence interval [CI] = 0.59–0.93, *P* = .009) after adjustment for clinical relevant variables. This benefit was consistent with that in the patients with NSTEMI who were ≥75 years old, had a CHA_2_DS_2_-VASc score of ≥2, and did not receive intra-aortic balloon pump insertion (aHR = 0.72, 95% CI = 0.53–0.98, *P* = .039; aHR = 0.73, 95% CI = 0.586–0.92, *P* = .006; and aHR = 0.71, 95% CI = 0.56–0.90, *P* = .004; respectively). However, DES placement had a neutral effect on the risk of new-onset AF in the patients with STEMI.

Compared with the use of BMS, the use of DES might reduce the risk of new-onset AF in patients with NSTEMI.

## Abstract 2

Supplemental Digital Content is available in the text

## 1 Introduction

Atrial fibrillation (AF) is a common arrhythmia that occurs during acute myocardial infarction (AMI), with the incidence reported to vary between 6% and 21%.^[1]^ Among all types of AF in the setting of AMI, new-onset AF carries an excess risk of short- and long-term adverse outcomes.^[2–4]^ Patients admitted for AMI who develop new-onset AF have an approximately 30% increased risk of a composite cardiovascular outcome (all-cause mortality, reinfarction, or ischemic stroke) within 90 days after discharge.^[4]^ Additionally, patients with new-onset AF occurring >30 days after receiving a diagnosis of AMI had an increased risk of death during a mean follow-up of 6.6 years (hazard ratio [HR] = 2.58, 95% confidence interval [CI] = 2.21–3.00)^[2]^ compared with patients without AF. These data indicate the importance of prevention of the occurrence of new-onset AF following AMI.

Prompt and early invasive treatment with percutaneous coronary intervention (PCI) for patients with AMI yields lower rates of adverse cardiovascular events than does medical treatment.^[5–8]^ Recent randomized clinical trials have demonstrated that the use of drug-eluting stents (DES) in patients with AMI treated invasively with PCI is associated with reduced rates of cardiac death, reinfarction, and target vessel revascularization compared with the use of bare-metal stents (BMS).^[9,10]^ Despite these positive findings, the association between DES use and the risk of new-onset AF following AMI is unknown. Additionally, whether the risk of new-onset AF associated with DES use differs between patients with ST-elevation myocardial infarction (STEMI) and those with non-ST-elevation myocardial infarction (NSTEMI) remains unclear.

Although the use of DES in patients with AMI is conventional, the cost of DES use is paid by patients whereas the cost of BMS use is fully covered by National Health Insurance (NHI) in Taiwan. This mentioned situation provided an opportunity to investigate the association between the risk of new-onset AF and stent types which was difficultly answered by randomized clinical trials. In the present retrospective cohort study, we investigated the effect of DES on the risk of new-onset AF in patients with STEMI and those with NSTEMI treated invasively with PCI at 1-year follow-up by using data from Taiwan's National Health Insurance Research Database (NHIRD).

## 2 Methods

### 2.1 Institutional review board and data set

This study was approved by the Institutional Review Board of Taipei MacKay Memorial Hospital (approval No. 17MMHIS161e). In this study, we used the NHIRD, a claim-based database that covers 99% of the residents in Taiwan under the purview of the NHI program. Because individuals are deidentified in the NHIRD data released to researchers, informed consent was waived under the full review process of the Institutional Review Board.

The NHIRD includes inpatient, outpatient, and prescription drug claims. Prescribed medications are classified according to the anatomical therapeutic chemical system, and the disease diagnosis is coded according to the International Classification of Diseases, Ninth Revision, Clinical Modification (ICD-9-CM). Death records from the National Death Registry are also linked to the NHIRD based on patients’ encrypted identifiers.^[11]^

### 2.2 Study cohort

**Figure 1:**
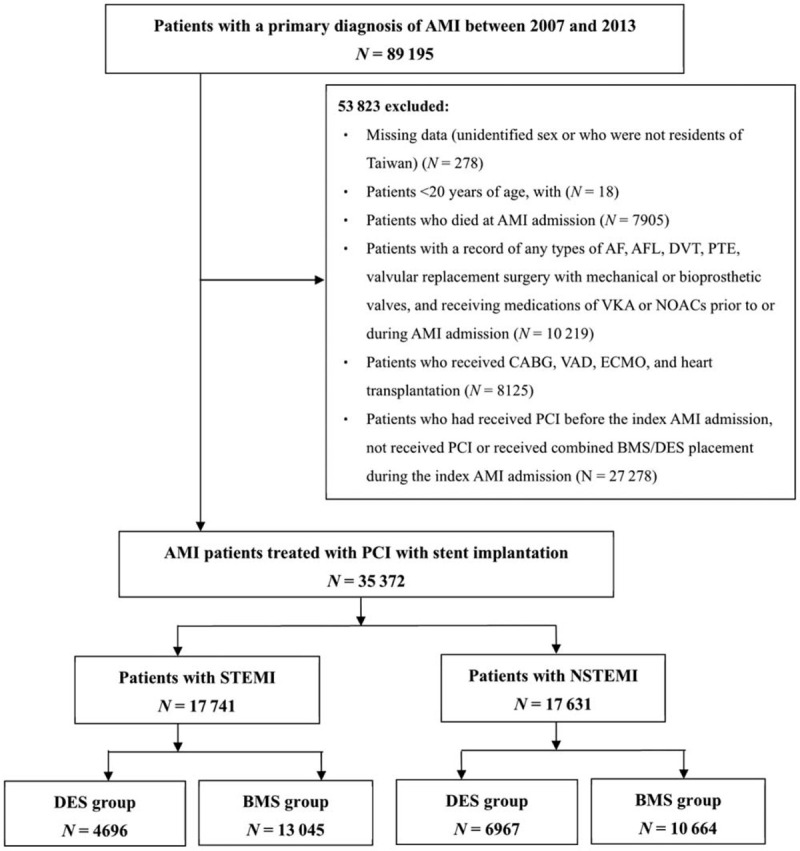
Patient selection process.

Within the retrospective cohort, we included patients who had received a primary diagnosis of AMI (ICD-9-CM code 410) based on the discharge claim between 2007 and 2013. The date of admission for AMI was considered the index of AMI. We excluded patients who were <20 years old; were not residents of Taiwan; had died at the index AMI admission; had preexisting AF or atrial flutter (AFL); had a record of AF or AFL at index AMI admission; and had received coronary artery bypass grafting, ventricular assist device support, extracorporeal membrane oxygenation, or heart transplantation during the study period. Patients who had medical conditions requiring anticoagulant treatment (eg, deep vein thrombosis, pulmonary thromboembolism, any type of AF or AFL, and valvular replacement surgery with mechanical or bioprosthetic valves) and received chronic vitamin K antagonist or nonvitamin K antagonist oral anticoagulants that may negatively influence the tolerance of dual antiplatelet therapy (DAPT) and preclude the physicians’ choice of DES were also excluded.^[12]^ In addition, patients who had received prior PCI before the index AMI admission and who did not receive PCI or stent implantation during the index AMI admission were excluded. Patients diagnosed as having 2 different subtypes of AMI, STEMI, and NSTEMI, were identified. Figure 1 presents the patient selection process. Patients who had received any DES and BMS implantation during PCI at their index AMI hospitalization constituted the DES and BMS groups, respectively.

### 2.3 Main outcome measures

The principal outcome in the present study was the new-onset AF requiring new prescriptions of vitamin K antagonist or nonvitamin K antagonist oral anticoagulants that can be defined from diagnostic claim of ICD-9-CM code of 427.31 and drug claims. The patients were followed up for 1 year, and the data of those who died or did not have the events of interest during the study periods were treated as censored cases.

### 2.4 Statistical analysis

Continuous variables are presented as mean ± standard deviation, and categorical variables are expressed as percentages. Comparisons between the baseline characteristics in the DES and BMS groups were performed using the chi-squared test for categorical variables and Student *t* test for continuous variables. The Kaplan–Meier method was used to report the cumulative incidence of events over time, and log-rank tests were applied to evaluate differences between the 2 groups. A multivariable Cox proportional hazard regression model was used to compare the risk of new-onset AF between DES groups and BMS groups after adjustment for clinical relevant variables, comorbidities, and prescribed medications. Clinical relevant variables included age, year of AMI admission, complex PCI procedures (ie, PCI for ≥2 vessels), and the use of intra-aortic balloon pump (IABP) counterpulsation during PCI. Patients’ comorbidities included diabetes mellitus, hypertension, hyperlipidemia, cerebrovascular disease, chronic kidney disease, congestive heart failure, chronic obstructive pulmonary disease or asthma, dementia, Parkinson disease, osteoarthritis, rheumatoid arthritis or rheumatism, and CHA_2_DS_2_-VASc scores (the sum of risk factors for congestive heart failure, hypertension, age ≥75 years, diabetes mellitus, stroke, vascular disease, age of 65 to 74 years, and sex category of women).^[13,14]^ Prescribed medications included angiotensin-converting enzyme inhibitors/angiotensin II receptor blockers, beta-blockers, nitrate, antiplatelets, statins, proton pump inhibitors, nonsteroidal anti-inflammatory drugs, and steroids. A subgroup analysis of patients who were ≥75 years old, had a CHA_2_DS_2_-VASc score ≥2, and had received treatment with or without IABP insertion during AMI admission was also performed. Due to enrichment of data from the NHIRD, no data were missing during adjustment for differences in baseline characteristics. The ICD-9-CM codes for disease diagnosis and the anatomical therapeutic chemical codes for medication are listed in Supplementary Table A. All analyses were performed using SAS/STAT 9.4 software (SAS Institute Inc., Cary, NC) and STATA 14 software (Stata Corp LP, College Station, TX). *P* < .05 was considered significant.

## 3 Results

### 3.1 Baseline characteristics

Among the patients admitted for AMI treated invasively by PCI with stent placement, 50.2% had STEMI and 33.0% had received DES placement. The rate of receiving DES placement was lower in the patients with STEMI than that in those with NSTEMI (26.5% vs 39.5%) (Table 1). In both STEMI and NSTEMI cohorts, the patients who had received DES placement were younger and had lower IABP use, fewer prior cerebrovascular disease events, more complex PCI procedures, and more prescriptions of angiotensin-converting enzyme inhibitors/angiotensin II receptor blockers, beta-blockers, nitrates, and statins compared with patients who had received BMS placement (Table 1).

**Table 1:**
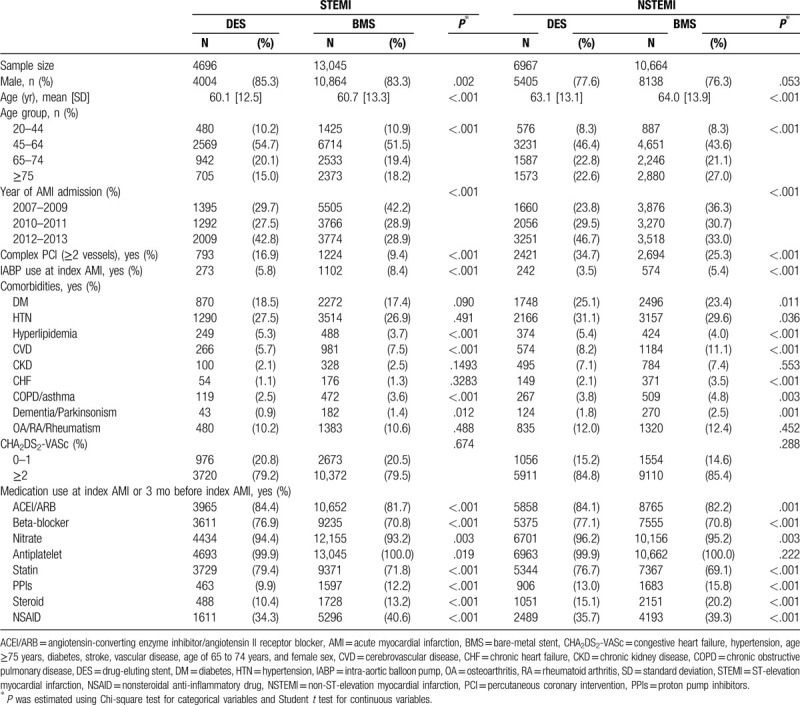
Basic characteristics of the patients with AMI treated with PCI and stent implantation.

### 3.2 Use of DES and risk of new-onset AF in patients with STEMI

**Figure 2:**
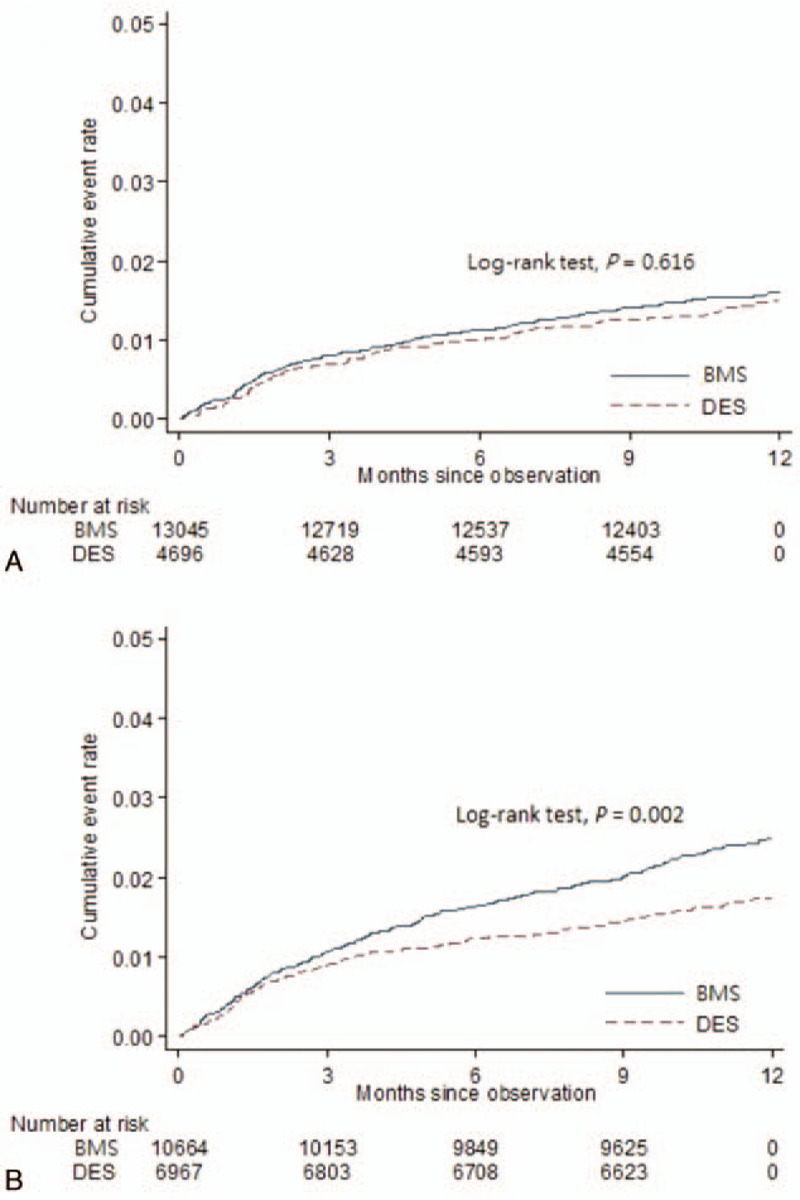
The Kaplan–Meier curve of the cumulative risk of new-onset AF in patients with STEMI (A) and in patients with NSTEMI (B) who had received DES or BMS placement within 1-year of follow-up after the index of AMI. AF = atrial fibrillation, AMI = acute myocardial infarction, BMS = bare-metal stents, DES = drug-eluting stent, NSTEMI = non-ST-elevation myocardial infarction, STEMI = ST-elevation myocardial infarction.

The cumulative incidence rate of new-onset AF in DES group was similar to that of the BMS group during the 1-year follow-up (Fig. 2A). Additionally, the incidence rates (per 100 person-year) of new-onset AF were also similar between the DES group (1.52, 95% confidence interval [CI] = 1.20–1.92) and BMS groups (1.64, 95% CI = 1.43–1.88) (Table 2). Any DES placement in patients with STEMI did not show a reduced risk of new-onset AF after adjustment for all variables compared with BMS placement (adjusted hazard ratio [aHR] = 1.00, 95% CI = 0.76–1.32, *P* = .989) (Table 2).

**Table 2:**
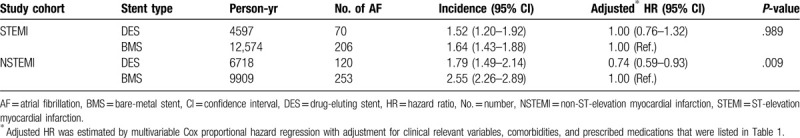
One-yr incidence (per 100 person-yr) and the risk of new-onset AF in patients with STEMI and patients with NSTEMI who had received DES or BMS placement.

### 3.3 Use of DES and risk of new-onset AF in patients with NSTEMI

The cumulative incidence rate of new-onset AF in the DES group was lower than that in the BMS group (Fig. 2B). Additionally, the incidence rates (per 100 person-year) of new-onset AF were lower in the DES group (1.79, 95% CI = 1.49–2.14) than that in the BMS group (2.55, 95% CI = 2.26–2.89) (Table 2). After adjusting for all variables, we found that any DES use in patients with NSTEMI was associated with a reduced risk of new-onset AF (aHR = 0.74, 95% CI = 0.59–0.93, *P* = .009) compared with BMS use (Table 2).

### 3.4 Subgroup analysis for patients who were ≥75 years of age, had a CHA_2_DS_2_-VASc score of ≥2, and who received treatment with or without IABP insertion

Of the NSTEMI patients aged ≥75 years, the incidence rates of new-onset AF were lower in the DES group than in the BMS group, with an aHR of 0.72 (95% CI = 0.53–0.98, *P* = .039). Additionally, the use of DES was associated with a reduced risk of new-onset AF (aHR = 0.73, 95% CI = 0.58–0.92, *P* = .006) in the patients with NSTEMI who had a CHA_2_DS_2_-VASc score of ≥2 (Table 3). However, the use of DES did not show a reduced risk of new-onset AF in the patients with STEMI who were ≥75 years old or had a CHA_2_DS_2_-VASc score of ≥2 (Table 3).

**Table 3:**
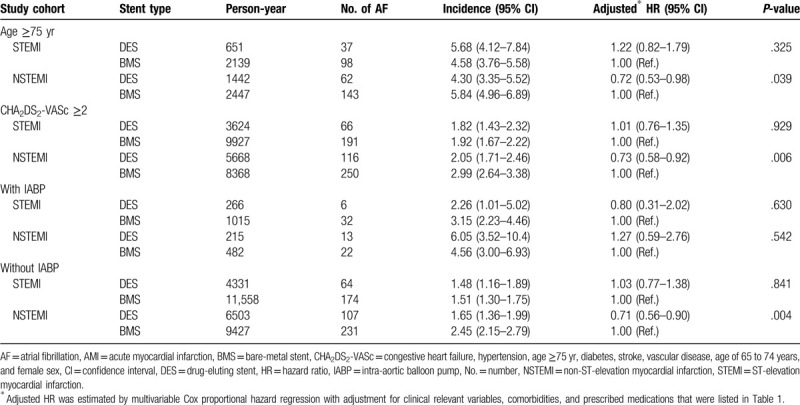
Subgroup analysis: 1-yr incidence (per 100 person-yr) and the risk of new-onset AF in patients with AMI who were ≥75 yr of age, had a CHA_2_DS_2_-VASc score of ≥2, and had received PCI with and without IABP insertion at the index of AMI admission.

In both patients with STEMI and NSTEMI who received IABP insertion during PCI, any DES use did not show a reduced risk of new-onset AF. Among the patients with NSTEMI who did not receive IABP insertion during PCI, DES placement was associated with a reduced risk of new-onset AF, with an aHR of 0.71 (95% CI = 0.56–0.90, *P* = .004) (Table 3).

## 4 Discussion

The current study revealed that the use of DES was associated with a reduced 1-year risk of new-onset AF in the patients with NSTEMI treated invasively with PCI. These benefits were also observed in the patients with NSTEMI who were ≥75 years old, had a CHA_2_DS_2_-VASc score of ≥2, and did not receive IABP insertion during PCI. However, the use of DES did not show a reduced risk of new-onset AF in the patients with STEMI treated with PCI.

Information on the risk of new-onset AF in patients with STEMI and NSTEMI treated with PCI with stent placement is limited. A retrospective observational study reported by Batra et al, who analyzed 155,071 patients admitted for AMI, showed that AF was recorded in 15.5% of patients, and the most common type of AF was new-onset AF with sinus rhythm at discharge.^[4]^ Additionally, patients with NSTEMI more often developed new-onset AF than patients with STEMI,^[4]^ as observed in the Global Registry of Acute Coronary Events study^[15]^ as well as in the present study. In this study, we observed a reduced risk of new-onset AF associated with DES use in the patients with NSTEMI treated invasively with PCI. The aforementioned results are consistent with those of the patients with NSTEMI who were ≥75 years old and had a CHA_2_DS_2_-VASc score of ≥2, as seen in previous studies.^[4,14,15]^

Data on DES use in patients with AMI who received IABP insertion is limited. The use of IABP counterpulsation does not improve clinical outcomes and may be only considered in critically ill patients who develop mechanical complications or refractory cardiogenic shock.^[5–8]^ A retrospective study that analyzed 652 patients with AMI and cardiogenic shock enrolled in the Intra-aortic Balloon Pump in Cardiogenic Shock II trial^[16]^ revealed that the 1-year risk of mortality and reinfarction of patients treated with DES was similar to those of patients treated with BMS.^[17]^ However, we did not observe that DES use was associated with a reduced risk of new-onset AF in patients with IABP insertion compared with BMS placement. This might be due to the relatively small numbers of patients with IABP insertion and hence, a statistically significant difference was not observed. This finding of the present study must be confirmed using an appropriately powered randomized clinical trial.

The current guidelines recommend that DAPT should be given for at least 12 months in patients with AMI regardless of DES or BMS implantation.^[18,19]^ The administration of triple therapy, including DAPT and an oral anticoagulant (OAC), is usually required for patients who develop new-onset AF following AMI to prevent the occurrence of ischemic stroke.^[20,21]^ However, triple therapy results in a 2- to 3-fold increase in bleeding complications compared with OAC therapy alone.^[18]^ A possible clinical implication of the present study is that DES placement be considered in patients with NSTEMI to reduce not only the risk of new-onset AF but also the requirement of OAC therapy and the occurrence of potential drug-related bleeding complications.

Any disturbance of atrial architecture potentially increases the susceptibility to AF. Atrial ischemia from coronary artery disease tends to increase the atrial pressure, cause atrial dilation, and result in structural and electrophysiological abnormalities, which promote abnormal impulse generation and propagation.^[20,21]^ A reduced risk of reinfarction and target vessel revascularization associated with DES use might lead to decreased recurrent atrial ischemia, limited atrial remodeling, and a reduced risk of AF formation. In the Swedish Coronary Angiography and Angioplasty Registry, PCI with DES placement was associated with a lower risk of stent thrombosis compared to that with BMS placement. Additionally, the superiority of DES over BMS for a lower risk of stent thrombosis became obviously in early months during the follow-up period in the Swedish Coronary Angiography and Angioplasty Registry data, which might also explain our findings.^[22]^ In the current study, patients with STEMI were mostly younger and had fewer comorbidities compared with patients with NSTEMI, as observed in previous studies.^[4,15]^ The favorable baseline characteristics of patients with STEMI themselves lead to a lowered risk of AF regardless of stent types.

The price of each DES paid by patients in Taiwan was approximately USD $2300 in the year of 2007 whereas the cost of BMS was fully covered by NHI. This mentioned gap in medical cost may introduce a bias in patients undergone either DES or BMS stent implantation, resulting in a difficulty to design a randomized study, especially in an emergent clinical situation with AMI. The strength of the present study is the nationwide population-based design with a large sample size and the use of real-world data, which can reflect the actual application of DES in patients with AMI. However, this study has some limitations. First, the NHIRD does not provide some clinical information, such as the extent of a patient's coronary artery disease, the angiographic findings of PCI, and some cardiac parameters (ie, left ventricular ejection fraction, left atrial size, and left ventricular end-diastolic pressure) that might influence the incidence of AF. Second, we did not analyze the differences between first-generation and newer-generation DES. In addition, we did not compare the effects of the different types of DES (ie, durable polymer DES and biodegradable polymer DES). Moreover, polymer-free drug coating stents and bioabsorbable vascular scaffolds were not commercially available in Taiwan until 2013 and were beyond the scope of the present study. Third, the baseline characteristics of patients might influence the choice of DES. For example, patients who had received DES implantation were more likely to have a higher socioeconomic status than those patients who had received BMS implantation because the medical cost of DES was not fully covered by NHI in Taiwan. This mentioned situation may somewhat influence the patients’ choice of stent types and needs to be addressed by further studies to investigate the benefits of DES compared with that of BMS. Besides, the patients with a high bleeding risk were more likely to receive BMS because of undetermined compliance of DAPT. We acknowledged that the socioeconomic status and bleeding risks would influence the choice of DES in the present study, as also seen in other research^[23]^; however, they might not be associated with the risk of AF. To estimate the association between the DES use and AF, this study used multiple regression methods to adjust potential confounding factors listed in Table 1. There were still some unobserved confounders that might bias the study results. Despite these limitations, our results presented a real-world data to address a clinical question that is difficultly answered by randomized trials. Nevertheless, the interpretation must be careful to consider the potential bias. Fourth, asymptomatic AF or AFL might lead to an underestimate of the measured outcomes. However, the misclassification bias should be non-differential between 2 exposure groups that was more likely to result in underestimating the effect of DES on the risk of AF. Finally, to clearly compare the effect of DES on the risk of new-onset AF, we only included the patients who were eligible for the current study, resulting in 60% of the initial population being excluded in the final analysis. Thus, the potential selection bias might exist. Moreover, the results cannot be generalized to all the population with AMI. Future prospective, randomized trials are warranted to confirm our findings.

## 5 Conclusions

In this population-based cohort study, the use of DES, compared with the use of BMS, is associated with a decreased risk of new-onset AF in the patients with NSTEMI treated with PCI at 1-year follow-up; these results are consistent with those for patients with a high risk to develop AF. Overall, our findings suggest that DES placement significantly hinders new-onset AF following NSTEMI, whereas the use of DES in the patients with STEMI results in a neutral effect compared with BMS placement.

## Author contributions

**Conceptualization:** Fa-Chang Yu.

**Data curation:** Fa-Chang Yu, Hung-Yi Liu, Chao-Feng Lin, Li-Nien Chien.

**Formal analysis:** Hung-Yi Liu, Chao-Feng Lin, Li-Nien Chien.

**Methodology:** Fa-Chang Yu, Chao-Feng Lin, Li-Nien Chien.

**Resources:** I-Ming Chen.

**Validation:** Ya-Hui Chang, Hung-Yi Liu.

**Visualization:** Ya-Hui Chang, I-Ming Chen.

**Writing – original draft:** Fa-Chang Yu, Chao-Feng Lin, Li-Nien Chien.

**Writing – review & editing:** Fa-Chang Yu, Ya-Hui Chang, I-Ming Chen, Hung-Yi Liu, Chao-Feng Lin, Li-Nien Chien.

## Supplementary Material

Supplemental Digital Content
