# Supplementary material for: Effect of drug-eluting stents on 1-year risk of new-onset atrial fibrillation in patients with acute myocardial infarction treated with percutaneous coronary intervention
Source: Medicine (Baltimore). 2020 Aug 21;99(34):e21885. doi: 10.1097/MD.0000000000021885 (PMC7447439; doi:10.1097/MD.0000000000021885)
Supplement: Supplemental Digital Content [file medi-99-e21885-s001.docx]

**Supplementary Table S1:** Disease diagnosis codes according to ICD-9-CM and ATC classification of medications

| **Comorbidities** | **ICD-9-CM** |
| --- | --- |
| AMI | 410 |
| STEMI | 410.1–410.6, 410.8 |
| Atrial fibrillation | 427.31 |
| Atrial flutter | 427.32 |
| DM | 250 |
| HTN | 401-405 |
| Hyperlipidemia | 272 |
| CVD | 430-438 |
| CKD | 585 |
| CHF | 428 |
| COPD | 491, 492, 494, 496 |
| Asthma | 493 |
| Dementia | 290–294 |
| Parkinsonism | 332 |
| OA | 715 |
| RA | 714 |
| Rheumatism | 729 |
| **Medications** | **ATC code** |
| ACEI/ ARB | C09AA C09BA C09BB C09CA C09DA C09DB C09DX |
| Beta-blocker | C07AA, C07AB, C07AG |
| Nitrate | C01DA, C01DX |
| Antiplatelet | B01AC |
| Statin | C10AA |
| PPI | A02BC |
| Steroid | H02, R03BA |
| NSAID | M01A M02A N02BA |
| NOACs | B01AE, B01AF |
| VKA | B01AA |

Abbreviation: ACEI/ARB=angiotensin-converting enzyme inhibitor/angiotensin II receptor blocker; AMI=acute myocardial infarction; ATC= Anatomical Therapeutic Chemical; CVD=cerebrovascular disease; CHF=chronic heart failure; CKD=chronic kidney disease; COPD=chronic obstructive pulmonary disease; DES=drug-eluting stent; DM=diabetes; HTN=hypertension; ICD-9-CM=International Classification of Diseases, Ninth Revision, Clinical Modification; OA=osteoarthritis, RA= rheumatoid arthritis; STEMI=ST-elevation myocardial infarction; NOACs=non-vitamin K antagonist oral anticoagulants; NSAID = nonsteroidal antiinflammatory drug; NSTEMI=non-ST-elevation myocardial infarction; PCI=percutaneous coronary intervention; PPIs = proton pump inhibitors; VKA=vitamin K antagonist.
